# Metformin suppresses retinal angiogenesis and inflammation *in vitro* and *in vivo*

**DOI:** 10.1371/journal.pone.0193031

**Published:** 2018-03-07

**Authors:** Jing Han, Yue Li, Xiuli Liu, Tongrong Zhou, Haijing Sun, Paul Edwards, Hua Gao, Fu-Shin Yu, Xiaoxi Qiao

**Affiliations:** 1 Department of Ophthalmology, Henry Ford Health System, Detroit, Michigan, United States of America; 2 Department of Ophthalmology, Tangdu Hospital, Fourth Military Medical University, Xi’an, Shaanxi, People’s Republic of China; 3 Departments of Ophthalmology and Anatomy and Cell Biology, Wayne State University, School of Medicine, Detroit, Michigan, United States of America; Children's Hospital Boston, UNITED STATES

## Abstract

The oral anti-diabetic drug metformin has been found to reduce cardiovascular complications independent of glycemic control in diabetic patients. However, its role in diabetic retinal microvascular complications is not clear. This study is to investigate the effects of metformin on retinal vascular endothelium and its possible mechanisms, regarding two major pathogenic features of diabetic retinopathy: angiogenesis and inflammation. In human retinal vascular endothelial cell culture, metformin inhibited various steps of angiogenesis including endothelial cell proliferation, migration, and tube formation in a dose-dependent manner. Its anti-angiogenic activity was confirmed *in vivo* that metformin significantly reduced spontaneous intraretinal neovascularization in a very-low-density lipoprotein receptor knockout mutant mouse (*p*<0.05). Several inflammatory molecules upregulated by tumor necrosis factor-α in human retinal vascular endothelial cells were markedly reduced by metformin, including nuclear factor kappa B p65 (NFκB p65), intercellular adhesion molecule-1 (ICAM-1), monocyte chemotactic protein-1 (MCP-1), and interleukin-8 (IL-8). Further, metformin significantly decreased retinal leukocyte adhesion (*p*<0.05) in streptozotocin-induced diabetic mice. Activation of AMP-activated protein kinase was found to play a partial role in the suppression of ICAM-1 and MCP-1 by metformin, but not in those of NFκB p65 and IL-8. Our findings support the notion that metformin has considerable anti-angiogenic and anti-inflammatory effects on retinal vasculature. Metformin could be potentially used for the purpose of treating diabetic retinopathy in addition to blood glucose control in diabetic patients.

## Introduction

Diabetic retinopathy (DR) is the leading cause of irreversible visual impairment and blindness at working age populations in the United States[1]. Clinical and basic research has revealed a complex pathogenesis of DR, including inflammation, angiogenesis, non-enzymatic glycosylation, redox injuries, and genetic predisposition factors [2]. Although most of these processes remains to be studied, there are two well described pathogenic features in the development of DR. One is leukocytes adhere to retinal vascular endothelium that occurs very early after the onset of diabetes, which subsequently triggers release of pro-inflammatory cytokines such as tumor necrosis factor-α (TNFα) and interleukins (ILs) [3, 4]. The other is retinal vascular endothelium angiogenesis, that attributes to vasculopathies including microaneurysms, hemorrhages, and neovascularization [2]. Current treatments of DR include panretinal photocoagulation, and intravitreal injection of anti-vascular endothelial growth factor agents and corticosteroids [5]. These treatments were able to retard severe DR progression in a subset of diabetic patients [6]. But they are invasive and traumatic, usually not applied until DR was symptomatic and vision loss was inevitable. Early and effective interventions that treat the initiative pathogenic changes of DR would be required to improve vision outcomes in diabetic patients.

Metformin was reported to effectively reduce blood glucose levels as early as 1922 by Werner E and Bell J [7]. Today, metformin is the first line medication for type 2 diabetes worldwide and the only medicine recommended for pre-diabetes patients due to its unsurpassed advantages of efficacy, safety, and inexpensiveness[8]. However, its mechanisms of action are yet to be fully understood. Research in recent years has revealed that metformin can improve cardiovascular outcomes in type 2 diabetic patients beyond its blood glucose control capacity [9–12]. We have observed that long-term oral metformin was associated with significantly reduced severity of DR in patients with type 2 diabetes[13]. Further, there were cumulative evidence that metformin could potently protect endothelial cells via anti-angiogenic, anti-inflammatory, and anti-oxidant mechanisms [14–17]. AMP-activated protein kinase (AMPK) was identified as one of the major signaling pathways that stimulated by metformin in the regulation of energy metabolism [18, 19] and endothelium functions[20]. Nonetheless, the role of metformin in diabetic retinal microvasculatures remains unclear.

In this study, we demonstrated that metformin directly inhibited angiogenesis of human retinal vascular endothelial cells (hRVECs), as well as prevented TNFα-induced upregulation of multiple inflammatory cytokines in hRVECs. These *in vitro* results were confirmed by *in vivo* evidence that metformin significantly suppressed intraretinal neovascularization (IRNV) and diabetes-induced retinal leukostasis in mice. AMPK signaling was partially involved in the anti-inflammatory actions of metformin.

## Materials and methods

### Cell culture and reagents

Primary human retinal microvascular endothelial cells (hRVECs) were purchased from Cell Systems (ACBRI 181, Kirkland, WA) and grown in an endothelial cell growth medium (EGM™-2 BulletKit, Lonza Inc., Allendale, NJ). Cells were maintained under standard incubation conditions according to the manufacturer’s instructions, and all experiments were done using cells between 3 to 10 passages. Metformin hydrochloride (Sigma-Aldrich, St. Louis, MO) stock solution was prepared at 1M for further dilution to final concentrations of 0~200 mM.

### Cell proliferation assay

Proliferation of hRVECs was determined by CellTiter 96® AQueous One Solution Reagent (Promega, Madison, WI) according to the manufacturer’s instructions. Briefly, hRVECs were seeded in 96-well plates at 4,000 cells per well in cell culture media containing 0~50 mM of metformin. After 24 hours incubation, the media of each well was removed. 80 μl culture media and 20μl reagent were added to each well and incubated for another 2 hours. The absorbance value at 490 nm was measured by a microplate reader (SpectraMax M2e, Molecular Devices LLC., Sunnyvale, CA).

### *In vitro* TUNEL assay

A TdT-mediated dUTP-fluorescein nick end-labeling (TUNEL) based In Situ Cell Death Detection Kit (Roche, Indianapolis, IN) was used to measure apoptosis of hRVECs. The cells were exposed to 0~200 mM metformin for 24 hours. Cells exposed to DNase I (Sigma-Aldrich, St. Louis, MO) (3000U/ml in 50 mM Tris-HCl, pH 7.5, 1mg/ml BSA) for 10 minutes at room temperature prior to TUNEL labeling procedure was included as positive control. A no-treatment group was used as negative control. The positively stained cells were counted under a fluorescence microscope (Leica DM IRB, Leica Microsystem Inc., Bannockburn, IL).

### Cell migration assay

Migration of hRVECs was assessed by a scratch-wound assay. Briefly, hRVECs at 100% confluence were maintained in 24-well plates. A linear scratch wound was created by scrapping across the confluent cells with a micropipette tip in each well. Images of the wound were captured with a digital camera (QCapture Pro 7, QImaging Corp., Surrey, BC, Canada) under a light microscope (Leica DM IRB, Leica Microsystems Inc., Bannockburn, IL). After incubation with 0~50 mM metformin for 12 hours, the scratch wound images were captured again. The gap area on each image was measured with Image-Pro Plus software (Media Cybernetics Inc., Silver Spring, MD). The results was displayed as the area occupied by migrating cells and scored as percentage of the control group. Three replicates were done in each individual experiment.

### Tube formation assay

HRVECs were seeded at 4000 cell/well in 96-well plates that pre-coated with Matrigel (BD Biosciences, San Jose, CA). 0~50 mM metformin were used to treat the cells for 6 hours. The capillary-like tube structure formed by hRVECs in each well was captured with a digital camera under a light microscope as described above. The total tube length in each well was quantified with Image-Pro Plus software (Media Cybernetics Inc., Silver Spring, MD).

### ELISA

After 12-hour pretreatment with 5 mM metformin, hRVECs were exposed to 2.5 ng/mL recombinant TNFα (Life technologies, Grand Island, NY) for another 12 hours. The protein concentrations of MCP-1 and IL-8 in hRVECs conditioned media were determined using ELISA kits (R&D Systems, Minneapolis, MN) following the manufacturer’s instructions.

### Western blot

Total protein of whole-cell or retinal tissue were extracted in RIPA buffer supplemented with protease inhibitors. Protein extracts were separated by 6–18% SDS–PAGE electrophoresis, and transferred to a polyvinylidene difluoride membrane. After blocking in 5% non-fat milk TBS/T buffer, blots were probed with different primary antibodies, including anti-NFκB p65 (Cat# 3033S, Cell Signaling Technology, Beverly, MA), anti-phosphorylated AMPKα (Cat# 2535S,Cell Signaling Technology, Beverly, MA, USA), anti-ICAM-1 (Cat# sc-7891,Santa Cruz Biotechnology, Dallas, TX), and anti-β-actin monoclonal antibody (Cat# sc-1616,Santa Cruz Biotechnology, Dallas, TX) at 4°C overnight. Blots were then incubated with HRP-conjugated secondary antibodies (Jackson ImmunoResearch, West Grove, PA) at room temperature for 1 hour, visualized by an enhanced chemiluminescence solution (Thermo Scientific, Rockford, IL), and quantified using Image Lab densitometry software of ChemiDoc™ MP System (Bio-Rad, Hercules, CA).

### Animals

All animals used in the study were maintained and treated with strict adherence to the guidelines for animal care and experimentation in the Association for Research in Vision and Ophthalmology Statement and with an animal protocol approved by the Henry Ford Health System Animal Care and Use Committee. Breeding pairs of mutant mice with targeted deletion of the very low density lipoprotein receptor (vldlr) gene and wild-type (WT) mice (C57BL/6) were obtained from Jackson Laboratory (Bar Harbor, ME). For all experimental procedures, mice were anesthetized by 80 mg/kg ketamine HCl and 16 mg/kg xylazine. For metformin treatment experiments, metformin hydrochloride oral solution (Ranbaxy Laboratories Inc, Jacksonville, FL) was given by daily gavage to mice (200 mg/kg/day) at specified age and duration. The dose of oral metformin gavage to mice was calculated according to clinically relevant human dose based on body surface area [21]. Every effort was made to avoid and minimize animal suffering.

### Quantification of IRNV

Metformin treatment was started at P10 (postnatal day 10), 4 days before the onset of spontaneous IRNV in *vldlr*^*-/-*^ mice [22]. After 10 days of metformin treatment, retina tissue of *vldlr*^*-/-*^ mice were collected for fluorescent-labeled isolectin staining of neovascularization according to previous reports [23, 24]. Briefly, the whole neural retina was dissected, fixed and stained with 1:200 diluted isolectin (Invitrogen, Life technology, Grand Island, NY) at 4°C overnight. A whole mount retinal preparation with photoreceptor side upward was made by 4–5 relaxing radial cuts and maintained in ProLong Gold antifade reagent (Invitrogen, Life technology, Grand Island, NY). The total number of IRNV in the entire retina was counted on images captured with QCapture Pro 7 (QImaging Corp., Surrey, BC, Canada) at low magnification (2.5x) focusing on the intraretinal space under an inverted fluorescence microscope (Leica DM IRB, Leica Microsystems Inc., Bannockburn, IL).

### Quantification of retinal leukostasis

Young adult C57BL/6 mice received daily intraperitoneal injection of 75 mg/kg streptozotocin (STZ) for 5 consecutive days. Blood glucose levels were checked two days later and monitored weekly thereafter. Only animals with consistently elevated glucose levels over 350 mg/dL were used as diabetic mouse. Metformin was given daily after 5 days of STZ injection for 10 weeks. The retinal adherent leukocytes were quantified by staining adherent leukocytes in the vasculature of a flat-mounted retina as described previously [25]. Under deep anesthesia, mice were first perfused with 10 mL of PBS to remove erythrocytes and nonadherent leukocytes. This was followed by perfusion of 10 mL fluorescein-isothiocyanate (FITC)-coupled concanavalin A lectin (Con A; Vector, Burlingame, CA) to label the adherent leukocytes and endothelial cells, and then by another 10 mL of PBS to remove residual unbound lectin. Retina was removed, fixed in 1% paraformaldehyde, and flat-mounted. The total number of Con A-stained leukocytes adhering to the retinal vascular wall was counted under a fluorescence microscope as described above.

### Statistical analysis

Results of quantitative studies were expressed as mean ± SEM of three independent experiments. Differences were assessed by Student's *t*-test or one-way ANOVA using PASW Statistics 18 (SPSS Inc., Chicago, IL). A value of *p* less than 0.05 was considered statistically significant.

## Results

### Metformin inhibits hRVEC angiogenesis without apoptosis

**Fig 1:**
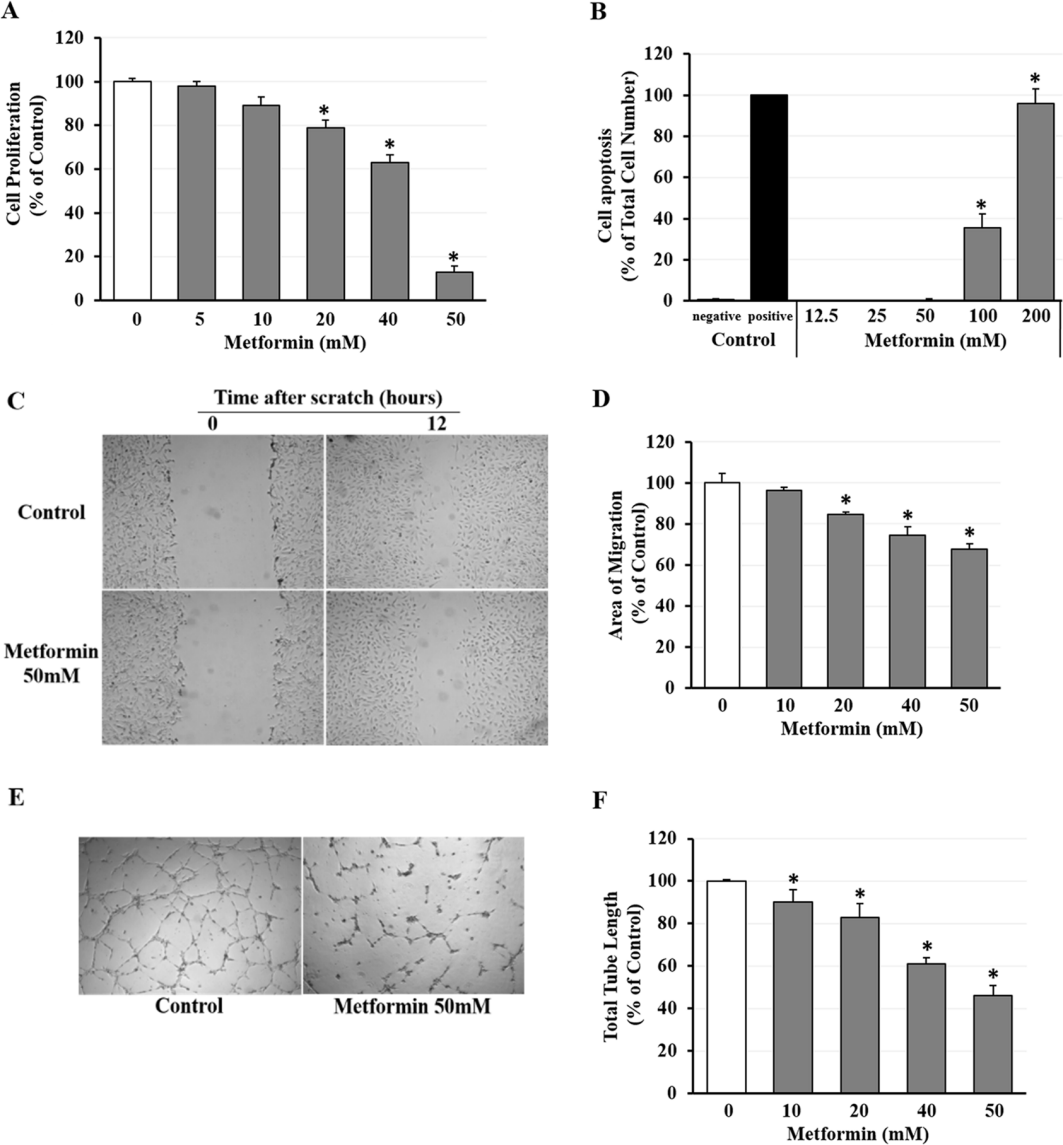
Effects of metformin on angiogenic activities and apoptosis of hRVECs. **(A)** Proliferation assay showed that metformin treatment at 5–50mM for 24 hours reduced hRVEC proliferation dose-dependently. **(B)** *In vitro* TUNEL assay revealed that exposure to 50 mM or lower metformin for 24 hours did not induce hRVECs apoptosis compared with negative control. Apoptosis of hRVECs was only seen when metformin dose reached 100 mM or higher. **(C)** Representative images and **(D)** quantitative analysis of scratch assay indicated a 12 hour treatment with metformin at 10–50 mM reduced hRVECs migration in a dose-dependent pattern. **(E)** Representative images and **(F)** quantitative analysis of hRVEC tube formation. After exposure to metformin at 0–50 mM for 6 hours, total length of capillary-like network formed by hRVECs decreased significantly and dose-dependently. Data were normalized to non-metformin treated control (in A, D, F) or DNase I treated positive control (in B) and presented as mean ± SEM (n = 3). * *p* < 0.05 versus non-metformin treated control.

The effects of metformin on angiogenic activities of hRVECs were summarized in Fig 1. A dose-dependent inhibition of hRVEC proliferation by metformin was evident by proliferation assay. Metformin at 20, 40, and 50 mM significantly suppressed proliferative activity of hRVECs by 21%, 37%, and 87%, respectively (Fig 1A; *p* < 0.05). To exclude a cytotoxic effect of metformin on hRVECs, TUNEL assay was performed. At 50 mM and below, metformin did not induce cell apoptosis after 24 hours. Notable cell apoptosis was only seen when metformin dose reached 100 mM or higher (Fig 1B; *p* = 0.01). Therefore metformin-induced reduction of hRVEC viability at and under 50 mM was not due to cell apoptosis.

The migratory capability of hRVECs under influence of metformin was determined by scratch-wound assay. The wound area of control groups was almost entirely covered by migrating hRVECs 12 hours after the scratch. In contrast, the wound area was significantly less covered by migrating cells with exposure to 50 mM metformin (Fig 1C). Quantitative analysis indicated a dose-dependent inhibitory effect of metformin on hRVEC migration. Exposure to 20, 40, and 50 mM metformin led to 14%, 25%, and 32% reduction of migrating cell-occupied area, respectively (Fig 1D; *p* < 0.05).

A matrigel based-tube formation assay revealed a similar but more potent suppressive effect of metformin on the capillary-forming capacity of hRVECs. While metformin at 10 mM failed to elicit any obvious change in the cell proliferation and migration, this concentration significantly reduced the total tube length formed by hRVECs (Fig 1F; *p* < 0.05). Treatment with 20, 40, and 50 mM metformin decreased the total length of capillary-like network by 17%, 39%, and 54%, respectively (Fig 1E and 1F; *p* < 0.05).

### Metformin inhibits development of IRNV in *vldlr*^*-/-*^ mice

**Fig 2:**
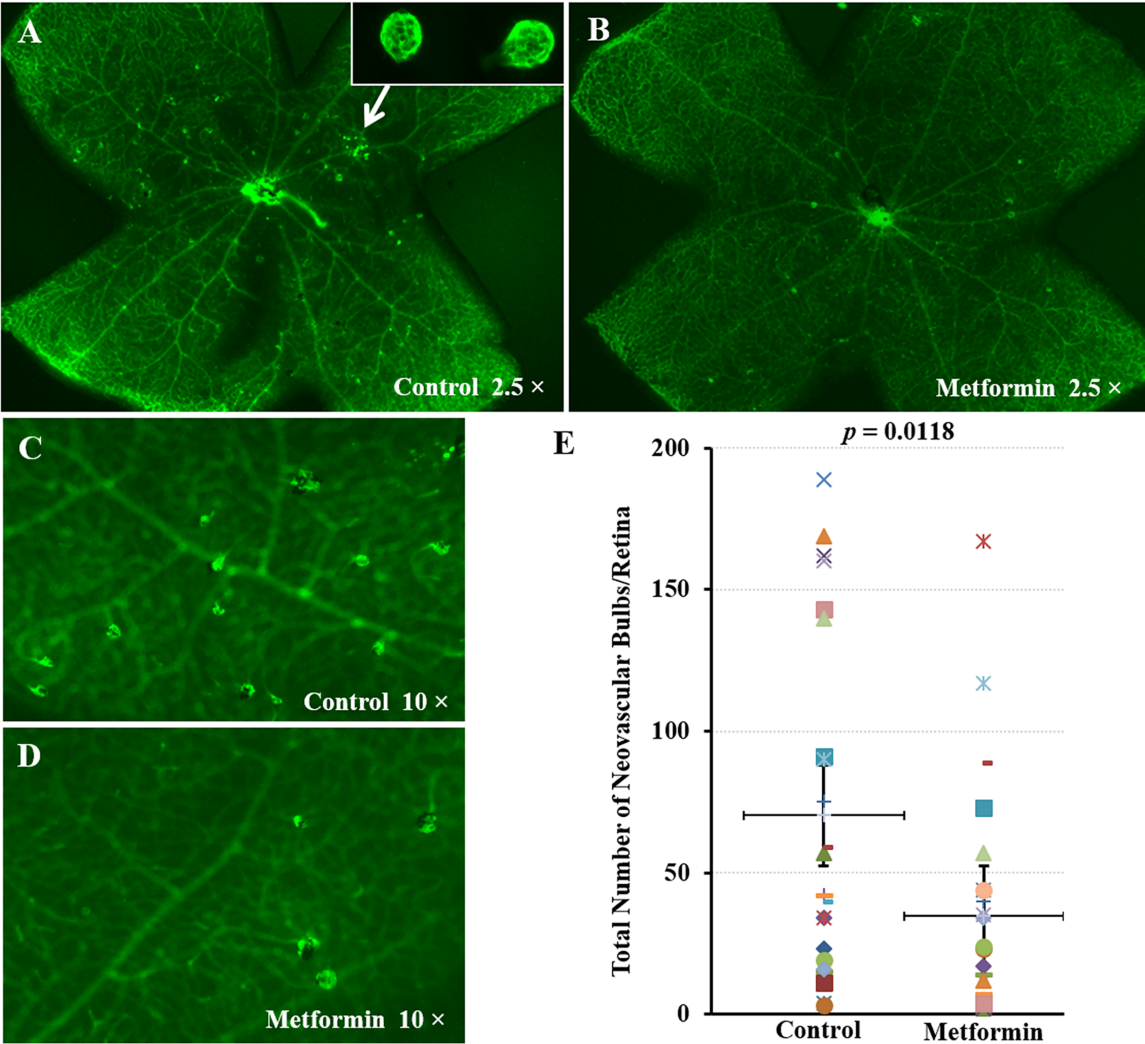
Effects of metformin on IRNV in *vldlr*^*-/-*^ mice. *Vldlr*^*-/-*^ mice were treated with metformin (200 mg/kg/day) or vehicle solution (control) by daily gavage from P10 to P21. Total number of IRNV sprouts was quantified at P21 in a whole mount retina after isolectin-FITC staining. **(A-D)** Representative images of isolectin stained retinal whole mounts from control (A, C) and metformin treated (B, D) P21 *vldlr*^*-/-*^ mice. **(E)** Quantitative measurement showed an over 50% reduction of the number of IRNV sprouts per retina in metformin treated group (n = 25) versus control group (n = 24) (*p* = 0.0118).

In order to investigate the influence of metformin in retinal angiogenesis *in vivo*, v*ldlr*^*-/-*^ mouse that develops spontaneous pathologic IRNV from P14 [22] was employed. *Vldlr*^*-/-*^ mice were treated with oral metformin at 200 mg/kg daily for 10 days from P10 to P21 to cover the initiating stage of IRNV development. Isolectin-FITC staining of retina whole mount revealed much less neovascular sprouts in *vldlr*^*-/-*^ mice treated with metformin versus those treated with vehicle control (Fig 2A–2D). In fact, metformin treatment reduced the total number of neovascular bulb by over 50% when compared with that of vehicle group (Fig 2E; *p* < 0.05).

### Metformin suppresses TNFα-induced inflammatory responses in hRVECs

**Fig 3:**
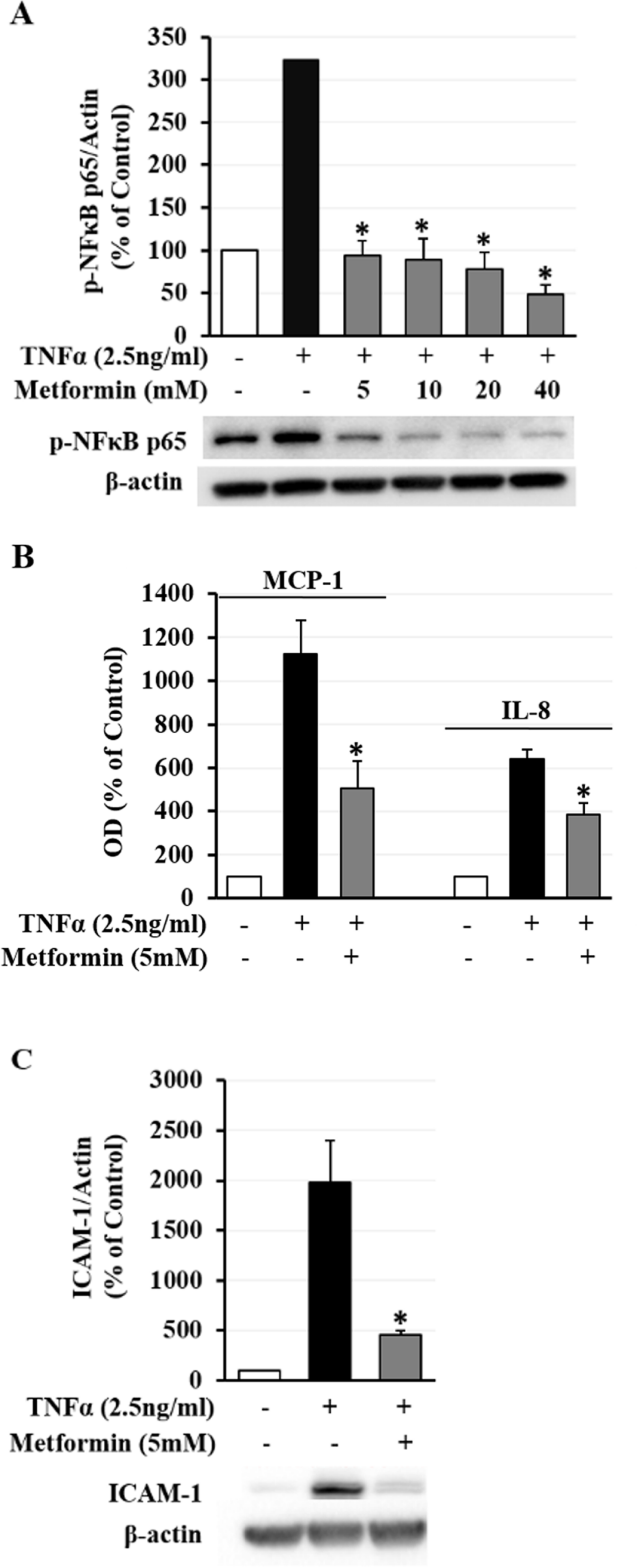
Effects of metformin pretreatment on NFκB, MCP-1, IL-8 and ICAM-1 in hRVECs with TNFα challenge. Cells were exposed to 2.5 ng/mL recombinant TNFα for 12 hours with or without 12-hour pretreatment of 5–40 mM metformin. The expression of NFκB p65 and ICAM-1 in hRVECs was measured by western blot. The concentration of MCP-1 and IL-8 in hRVEC culture media was determined using ELISA kits. (A) Representative blots and densitometry analysis revealed that TNFα upregulated expression of NFkB p65 in hRVECs, which was dramatically inhibited by metformin at 5–40 mM. (B) ELISA assay revealed that 5 mM metformin significantly blocked TNFα-induced secretion of MCP-1 and IL-8. (C) Representative blots and band intensity measurement of ICAM-1 immunoblot showed that metformin at 5 mM markedly prevented TNFα-induced upregulation of ICAM-1 in hRVECs. Data were normalized to control (without metformin or TNFα) and presented as mean ± SEM (n = 3). * *p* < 0.05 versus TNFα only group.

TNFα is a known initiator of inflammatory cascade that strongly correlates with the severity of DR[26]. In this study, we have found that 2.5 ng/mL of TNFα dramatically elevated the expression of NFκB p65, ICAM-1 and production of MCP-1, IL-8 by hRVECs (Fig 3A–3C). Strikingly, such strong upregulation of NFκB p65 was completely blocked by metformin pretreatment at a dose of 5 mM (Fig 3A; *p* < 0.001). Pretreatment with 5 mM metformin also significantly attenuated TNFα-induced production of MCP-1 and IL-8 (Fig 3B; *p* < 0.001). The upsurge expression of ICAM-1 was diminished by metformin treatment as well (Fig 3C; *p* < 0.001).

### Metformin suppresses diabetes-induced retinal leukocyte adhesion *in vivo*

**Fig 4:**
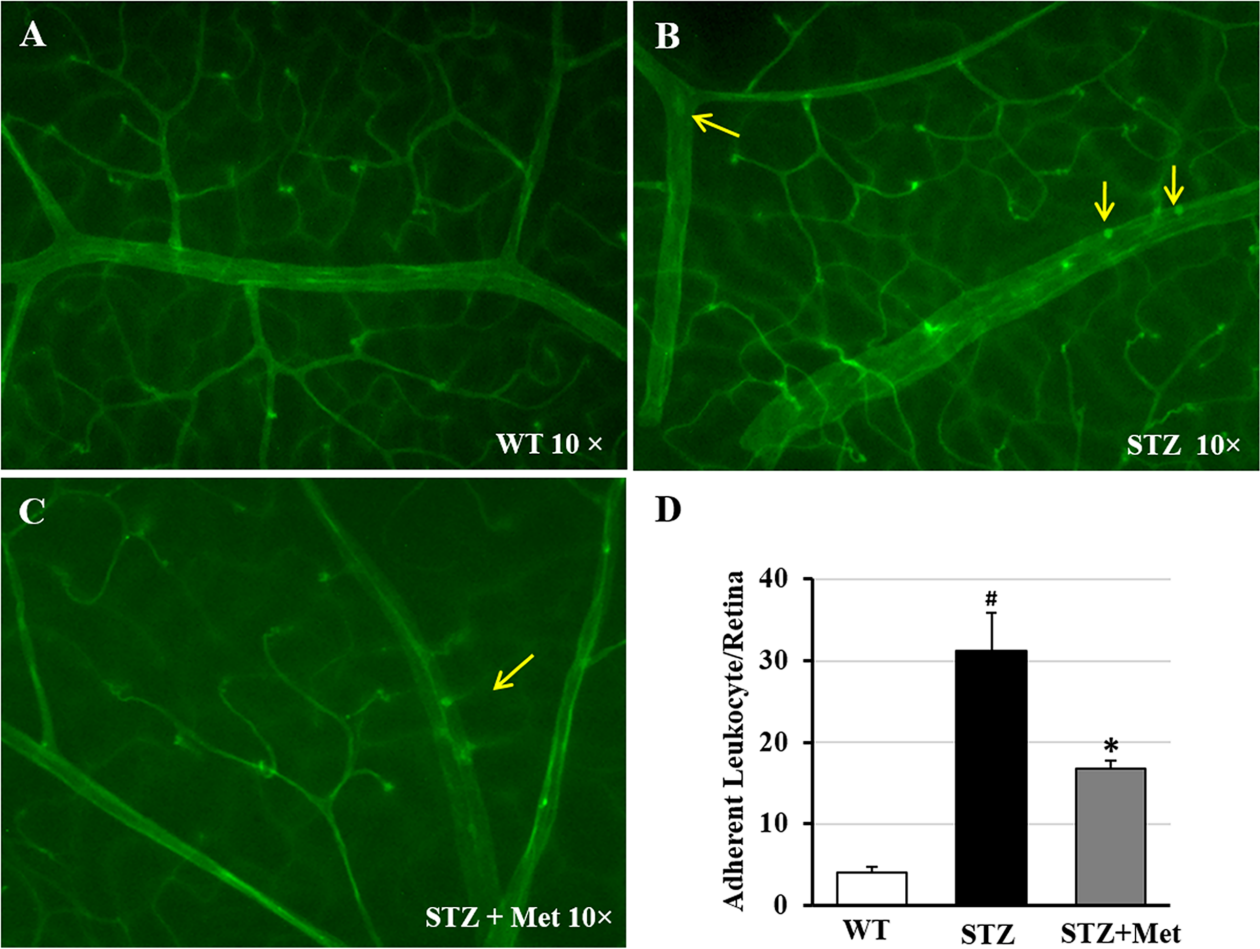
Effects of metformin on retinal leukostasis in STZ-induced diabetic mice. STZ-induced diabetic mice were treated with metformin (200 mg/kg/day) or balanced salt solution by daily gavage after 5 days of STZ injection for 10 weeks. Age-matched nondiabetic mice were used as wild type control. Retinal leukostasis was examined by perfusion labeling with FITC-coupled Con A. **(A-C)** Representative images of retinal flat mounts from wild type mice (A) and STZ-induced diabetic mice with (C) or without metformin treatment (B). Arrows indicated adherent leukocytes in retinal vasculature. **(D)** Quantification analysis showed a significant higher number of leukocytes per retina in STZ-induced diabetic mice versus wild type control, which was significantly reduced by metformin treatment. WT, wild type mice. STZ, STZ-induced diabetic mice treated with balanced salt solution. STZ+Met, STZ-induced diabetic mice treated with metformin. ^#^ *p* < 0.05 versus wild type mice; * *p* < 0.05 versus STZ-induced diabetic mice treated with vehicle solution.

Leukocyte adhesion to the retinal vasculature is an early sign of inflammatory insults in diabetes and implicated in endothelial cell injury and capillary dysfunctions [27]. In age-matched control mice, FITC-labeled leukocytes were barely seen in the retinal vessels (Fig 4A). In contrast, a number of leukocytes were clearly visible to adherent to the retinal vessel walls in STZ-induced diabetic mice after 10 weeks of diabetes (Fig 4B). In diabetic mice that received 10 weeks of oral metformin, much less adherent leukocytes were observed in the retinal vasculature (Fig 4C). Quantitative analysis revealed that diabetes led to an over 7-fold increase of adherent leukocytes in the large retinal vessels (Fig 4D; *p* < 0.001), which decreased by nearly half by metformin treatment (Fig 4D; *p* < 0.05).

### AMPK activation partially mediates metformin’s anti-inflammatory effects

**Fig 5:**
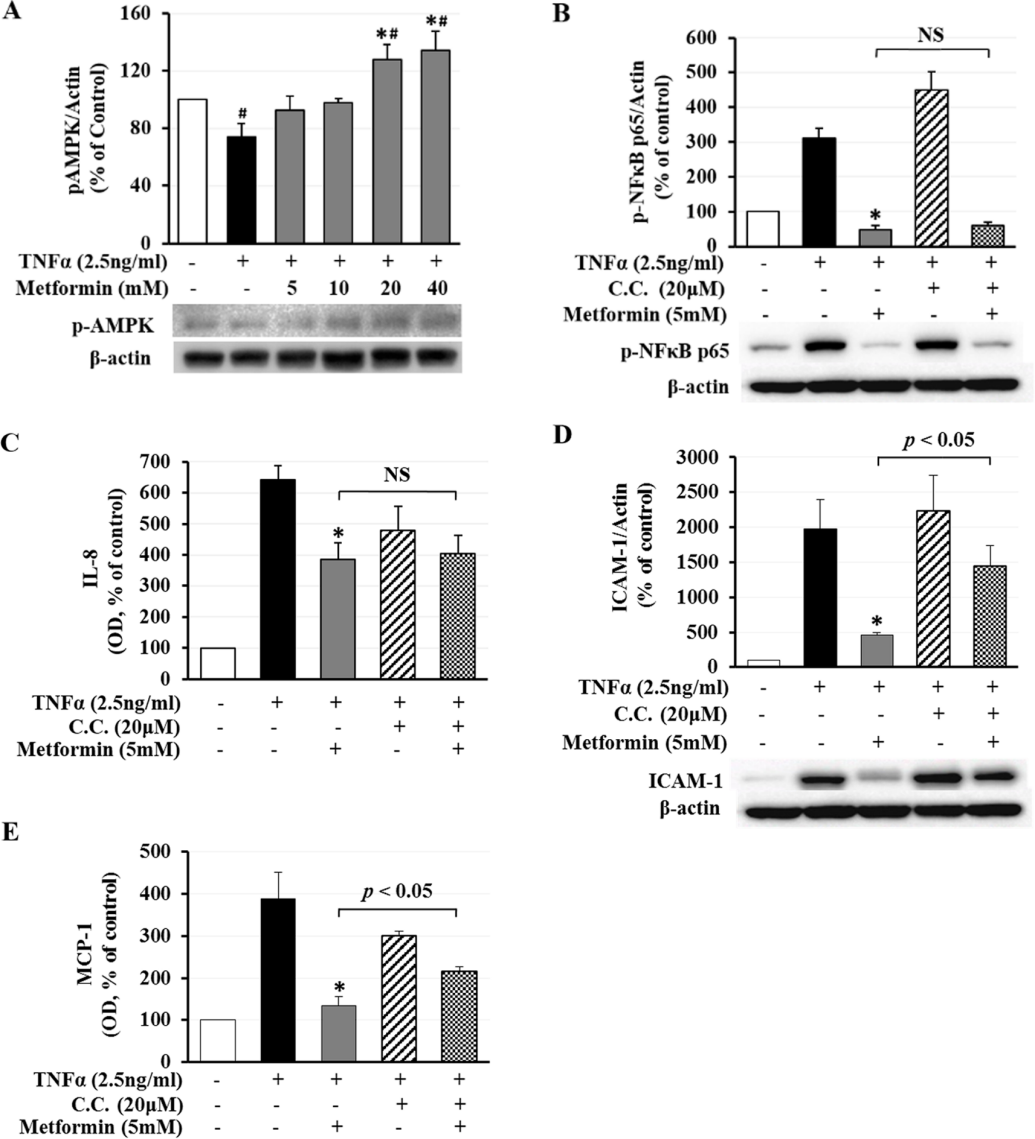
AMPK inhibition in metformin’s effects on NFκB, MCP-1, IL-8 and ICAM-1 in hRVECs. HRVECs were pretreated with metformin, then exposed to TNFα as described in Fig 3. A selective AMPK inhibitor, compound C, was co-administrated to evaluate the role of AMPK signaling in metformin’s influence on hRVECs. **(A)** Representative images and densitometric analysis of pAMPKα immunoblot in hRVECs. TNFα markedly reduced the level of pAMPKα, which was impeded by pretreatment of metformin dose-dependently. **(B)** Western blot of NFkB p65 and **(C)** ELISA of IL-8 revealed no obvious changes were caused by addition of compound C in metformin regiment in the presence of TNFα. **(D)** Western blot of ICAM-1 and **(E)** ELISA of MCP-1 showed that compound C significantly blocked the inhibition of ICAM-1 and MCP-1 by metformin. Data were normalized to control (without metformin and TNFα) and presented as mean ± SEM (n = 3). ^#^ *p* < 0.05 versus control. * *p* < 0.05 versus TNFα-only group.

Metformin is known to activate the key cellular metabolic regulator AMPK in many tissues, including hRVECs (S1 Fig). In this study, phosphorylated AMPKα (pAMPKα) was markedly reduced by TNFα challenge (Fig 5A, *p* < 0.05). Pretreatment with 5 or 10 mM metformin completely reversed this decrement of pAMPKα. Moreover, metformin at higher doses of 20 or 40 mM further elevated the levels of pAMPKα exceeding the basal level (Fig 5A; *p* < 0.05) in hRVECs. The highest level of pAMPKα was nearly double of that in the TNFα alone group. These results demonstrate that metformin not only can counteract TNFα insults and restore pAMPKα level, but also further activate AMPK dose-dependently.

As shown in Fig 3, metformin pretreatment significantly inhibited several inflammatory molecules that stimulated by TNFα in hRVECs, including NFκB, ICAM-1, MCP-1, and IL-8. To investigate the role of AMPK signaling in metformin-mediated anti-inflammatory responses in hRVECs, a selective AMPK inhibitor, compound C, was used. When compared with TNFα only group, addition of compound C did not cause significant changes on the levels of inflammatory molecule IL-8, ICAM-1, and MCP-1 (Fig 5C–5E; *p* > 0.05), but significantly augmented the expression of pNFκB (Fig 5B; *p* < 0.05). When compared with metformin plus TNFα group, addition of compound C showed different effects on these factors. The suppression of NFκB p65 (Fig 5B) and IL-8 (Fig 5C) by metformin was not altered by addition of compound C. On the other hand, compound C blocked a large portion of metformin-caused decrement in ICAM-1 expression (Fig 5D; 70%; *p* < 0.05 versus metformin plus TNFα group). Compound C also partially reversed the inhibition effect of metformin on MCP-1 concentration (Fig 5E; 50%; *p* < 0.05 versus metformin plus TNFα group). These results indicate that AMPK signaling pathway is involved in the regulation of ICAM-1 and MCP-1 by metformin in hRVECs, but not in that of NFκB and IL-8.

We have also tested the effect of AICAR, a direct AMPK activator, on TNFα-induced inflammatory responses in hRVECs to compare with metformin. As shown in S2 Fig, AICAR at 0.5 and/or 1 mM decreased TNFα-induced upregulation of ICAM-1 (S2A Fig) and MCP-1 (S2B Fig). The effects are similar to 5~10 mM metformin. However, unlike metformin, the effect of AICAR on pNFκB level was minimal (S2C Fig). These results revealed that while both AICAR and metformin elicit anti-inflammatory effects, however there are some differences between these two AMPK activators. It can be explained by that AICAR is a direct AMPK activator by binding to AMPKγ subunit, while metformin is an indirect activator by inhibiting Complex I of the mitochondrial respiratory chain.

## Discussion

Clinical management of DR remains a growing challenge with global epidemic of diabetes. It is critical to treat the patient before the retinopathy progress to vision-threatening stage. Screening and early detection programs for DR has been recommended by American Diabetes Association (ADA)[28] and the American Academy of Ophthalmology (AAO)[29]. However, it was estimated more than 50% of diabetic patients failed to comply with annual screens or receive timely laser photocoagulation in the United States[30, 31]. An optimized therapy for DR should be able to cover the asymptomatic early stages of the disease to prevent severe DR from happening. Metformin could potentially serve this purpose due to its well-established safety and efficiency profile in early treatment of diabetes, as well as pleiotropic effects in cardiovascular and nephrovascular system [9, 11, 32].

Angiogenesis and inflammation are two major components that contribute to the pathogenesis of DR from the early stages. Since there is no good rodent model available so far which could mimic all key features in DR development, we used different *in vitro* and *in vivo* models characterized by either angiogenesis or inflammation to study the effects of metformin on each of these two components respectively. In this study, metformin exhibited significant anti-angiogenic actions by inhibiting proliferation, migration and tube formation of hRVECs *in vitro*, as well as reducing a large portion of neovascular sprouts in *vldlr-/-* mice with spontaneous IRNV. Metformin also elicited remarkable anti-inflammatory effects by suppressing levels of several inflammatory molecules including NFκB, ICAM-1, MCP-1 and IL-8 in TNFα-stimulated hRVECs, and reducing retinal leukostasis in STZ-induced diabetic mice. Activation of AMPK by metformin was involved in its regulation of ICAM-1 and MCP-1, but not of NFκB and IL-8.

### Metformin impedes retinal angiogenesis

The earliest clinically noticeable signs of DR include microaneurysms, which are caused by localized proliferation of capillary endothelial cells[33], and loss of pericytes[34]. In the late stage, endothelial proliferation and subsequent neovascularization were found directly related to occurrence of PDR [35, 36]. Previous reports have found that metformin suppressed angiogenesis in some tissues but not in others. For example, metformin inhibits tube-formation of human dermal microvascular endothelial cells [14] and human umbilical vein endothelial cells (HUVEC) [37, 38]. On the other hand, metformin improves angiogenesis in ovarian [39] and brain [40]. One possible explanation is that metformin regulates different angiogenesis-related genes in a tissue-specific manner [14, 38, 41]. Our study revealed a broad spectrum anti-angiogenic activities of metformin in hRVECs. Therefore, we were eager to test if metformin could be used to prevent retinal neovascularization *in vivo*. *Vldlr*^*-/-*^ mouse is an ideal animal model for the study of retinal angiogenesis without significant features of retinal or systemic inflammation. *Vldlr*^*-/-*^ mice develop spontaneous IRNV starting at P14, which then progresses to subretinal neovascularization and choroidal neovascularization, and mimics the key pathological features of retinal angiomatous proliferation in human [22, 23, 42, 43]. In this study, oral metformin was applied before the onset of IRNV in order to evaluate a possible preventive action on angiogenesis. Although metformin did not completely abolish the growth of IRNV, it effectively reduced IRNV by over 50%. In contrast to our study, Kim et al [44] observed no significant effect of metformin on retinal neovascularization in a high-fat diet (HFD)-induced diabetic mouse model. In Kim’s study, metformin was orally administrated not as a preventive treatment, but after 2 months of the HFD regimen. The discrepancy could either come from different models used or from the timing of the treatment. Metformin is known preventive medicine which may not be able to reverse established cellular damage. On the other hand, Joe SG et al [37] reported blockade of retinal neovascularization by metformin in oxygen-induced retinopathy (OIR) mice. In addition, the anti-angiogenic activity of metformin was also supported by reports of cancer research and was believed to be independent of glycemic control[45].

#### Metformin inhibits retina inflammation, partially through activation of AMPK

Leukocytes adherent to the endothelium of retinal vasculature and subsequent release of inflammatory molecules are critical events that disrupt the blood retinal barrier in the early stage of DR [3, 46]. NF-κB, MCP-1, IL-8, and ICAM-1 are among the known pro-inflammatory cytokines involved in this process [46]. MCP-1 and IL-8 participated in monocyte recruitment and adhesion to endothelial cells [47, 48]. The adhesion molecule ICAM-1 was found upregulated in the retina of diabetic patients [49]. Pharmacologically blockade [50] or genetically knockout [3] of ICAM-1 had a protective effect against leukostasis and vascular leakage in early DR. These molecule are plausible therapeutic targets of anti-inflammation in DR. Here we demonstrated that pretreatment of metformin significantly reduced the levels of NFκB p65, ICAM-1, MCP-1, and IL-8 in TNFα-stimulated hRVECs, as well as leukostasis in the retinal vessels of diabetic mice. The inhibition of these inflammatory cytokines might as well play a role in metformin’s anti-angiogenesis actions, which merits further investigation. These findings showed potential benefits of metformin treatment in DR by inhibiting inflammatory responses. Similar anti-inflammatory effects of metformin have been reported in HUVECs and vascular smooth muscles [51–53]. Further, metformin was associated with decreased serum soluble vascular cell-adhesion molecule-1 (sVCAM-1) and soluble ICAM-1 levels in patients with type 2 diabetes during a follow up of 4 years [54]. All these evidence point to a promising role of metformin in treating DR via its pleiotropic effects on endothelial functions and inflammatory responses.

Although the anti-inflammatory effects of metformin has been described in different types of tissues, its mechanism is still not fully understood. Some studies suggested activation of AMPK constitutes the critical mechanisms underlying the pleiotropic effects of metformin [52, 55, 56]. On the other hand, inactivated AMPK signaling was associated with inflammatory reactions in DR [57]. The results of our study indicate that metformin increases the level of p-AMPK in hRVECs. Further, downregulation of ICAM-1 and MCP-1 by metformin was counteracted by AMPK inhibitor, compound C. However, compound C showed no effects on the levels of NFκB and IL-8. Our findings suggest that the anti-inflammatory effect of metformin is mediated by both AMPK dependent and AMPK independent mechanisms in hRVECs.

In our *in vitro* study, inhibition of hRVEC angiogenesis occurred at a dose range of 10–50 mM while 5 mM metformin effectively prevented inflammatory responses to TNFα. This dose range is adopted based on our preliminary experiments as well as similar studies in HUVECs [38] and hepatocytes [58]. In type 2 diabetic patients, therapeutic plasma concentration varies significantly despite a clear dosage guide of 1–2.5 g/day for type 2 diabetes. A recent review of 120 publications reported 65 different concentrations with the highest boundary at 1800 mg/L (10.91 mM) [59]. Although on the high end, the dose used in this study is within the bulk range of plasma concentrations in human and other in vitro studies. No significant cellular stress or apoptosis was observed at this dose range in hRVECs.

## Conclusions

In the current study, metformin has potent anti-angiogenic and anti-inflammatory effects on hRVECs. Agree with the *in vitro* findings, metformin also reduced retinal neovascularization in *vldlr*^*-/-*^ mice and suppressed leukostasis in STZ-induced diabetic mice. These results show that metformin is capable of targeting key pathogenic components in DR, which strongly implicate clinical usage of metformin in the treatment of DR by inhibiting local inflammation and angiogenesis. Since metformin is known to be well-tolerated, inexpensive, and efficient in treating early diabetes, our findings brought up an appealing concept of “repurposing” the drug for DR prevention and treatment.

## Supporting information

S1 Fig Metformin on pAMPK level in hRVECs. Western blot analysis revealed that metformin treatment at 5, 10, and 20 mM dose-dependently increased the levels of pAMPK in hRVECs.
(TIF)

S2 Fig Effects of metformin and AICAR on TNFα-induced inflammatory molecule productions in hRVECs. AICAR at 0.5 and/or 1 mM decreased TNFα-induced upregulation of ICAM-1 (A) and MCP-1 (B). The effects are equivalent to 5~10 mM metformin. However, unlike metformin, AICAR had minimal effect on pNFκB level (C). *p < 0.05 when compared to TNFα alone group.
(TIF)

S1 File NC3Rs ARRIVE guidelines Checklist. The Section and Paragraph location is listed for each item in the Checklist.
(PDF)
