# Supplementary figures and images for: Metformin suppresses retinal angiogenesis and inflammation in vitro and in vivo
Source: PLoS One. 2018 Mar 7;13(3):e0193031. doi: 10.1371/journal.pone.0193031 (PMC5841739; doi:10.1371/journal.pone.0193031)

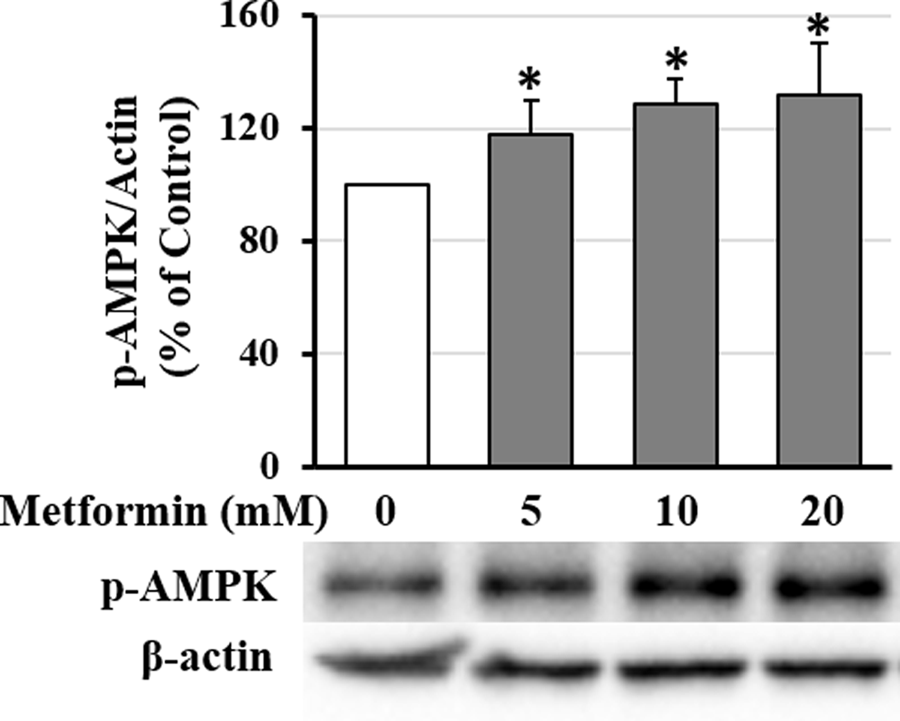

Supplement: S1 Fig — Western blot analysis revealed that metformin treatment at 5, 10, and 20 mM dose-dependently increased the levels of pAMPK in hRVECs. (TIF) [file pone.0193031.s001.tif]

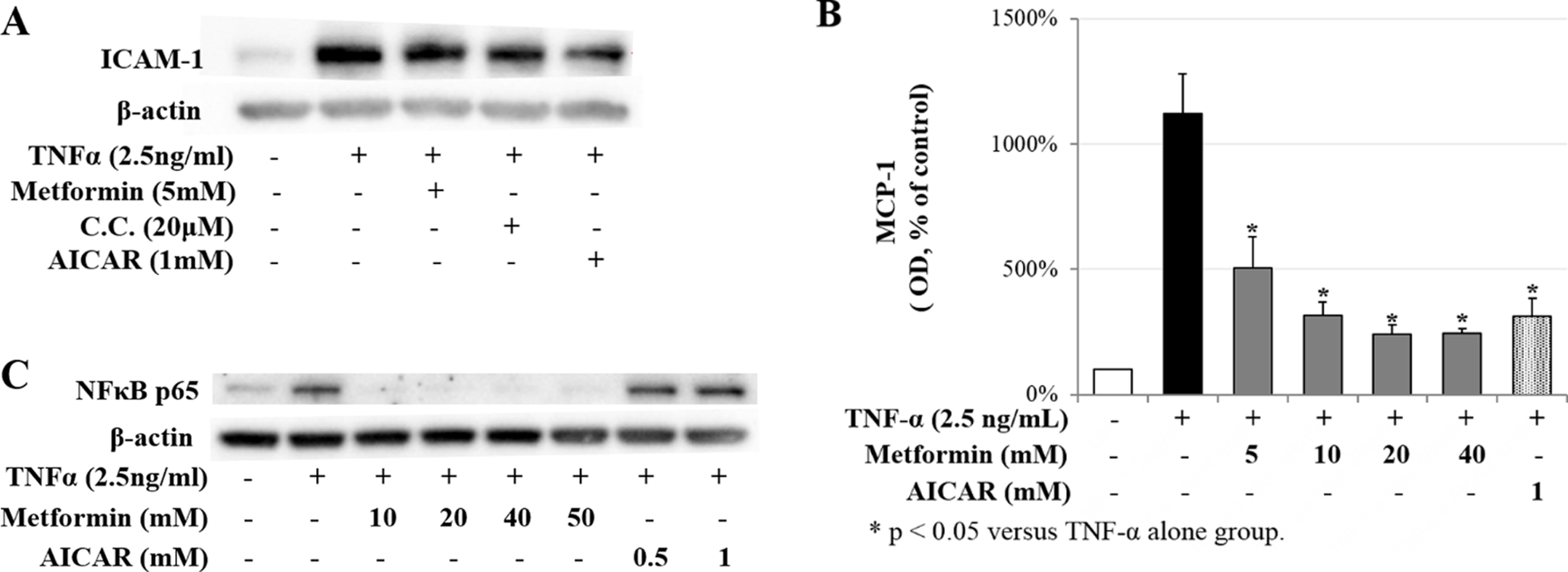

Supplement: S2 Fig — AICAR at 0.5 and/or 1 mM decreased TNFα-induced upregulation of ICAM-1 (A) and MCP-1 (B). The effects are equivalent to 5~10 mM metformin. However, unlike metformin, AICAR had minimal effect on pNFκB level (C). *p < 0.05 when compared to TNFα alone group. (TIF) [file pone.0193031.s002.tif]
